# Investigating the impact of value congruence on work engagement in EFL teachers: the role of teacher enthusiasm

**DOI:** 10.3389/fpsyg.2023.1264126

**Published:** 2023-10-12

**Authors:** Jing Cao, Weijing Zhang

**Affiliations:** ^1^School of Foreign Languages, North China Institute of Science and Technology, Tangshan, China; ^2^Department of Humanities and Law, Hebei University of Engineering, Handan, China

**Keywords:** value congruence, EFL teachers, work engagement, teacher enthusiasm, structural equation modeling, job satisfaction, well-being

## Abstract

**Introduction:**

This research aimed to investigate the influence of value congruence on EFL (English as a Foreign Language) teachers’ work engagement, with a focus on the mediating role of teacher enthusiasm.

**Methods:**

A sample of EFL teachers (*N* = 453) in China participated in the study. Data were collected using self-report measures, including the Value Congruence Scale, Teacher Enthusiasm Scale, and Work Engagement Scale. Structural equation modeling was employed to analyze the data and test the proposed model.

**Results:**

The results revealed that value congruence had a significant positive direct effect on work engagement. Furthermore, teacher enthusiasm was found to mediate the relationship between value congruence and work engagement.

**Discussion:**

These findings suggest that when EFL teachers perceive a congruence between their personal values and the values upheld by their educational institutions, they are more likely to experience higher levels of work engagement, and this relationship is partially explained by their level of enthusiasm. The study contributes to the understanding of the factors that influence EFL teachers’ work engagement and highlights the importance of value congruence and teacher enthusiasm in fostering a positive work environment. These findings have implications for the development of interventions and practices aimed at enhancing EFL teachers’ well-being and job satisfaction.

## 1. Introduction

The field of education has witnessed growing interest in understanding the factors that contribute to teachers’ work engagement and well-being. Work engagement, defined as a positive psychological state characterized by vigor, dedication, and absorption in one’s work, has been linked to various positive outcomes, including job satisfaction, performance, and student achievement (Schaufeli et al., 2002; Bakker and Demerouti, 2008; Pérez-Fuentes et al., 2019). As teachers play a critical role in shaping students’ learning experiences, investigating the factors that promote their work engagement is crucial for both individual well-being and educational effectiveness. As a crucial psychological variable influencing success, motivation, and fulfillment, teachers’ work engagement refers to an enduring, optimistic, affective-motivational condition distinguished by vitality, commitment, and immersion (Høigaard et al., 2012; Perera et al., 2018; Pérez-Fuentes et al., 2018; Xiao et al., 2022). According to Cardwell (2011), work engagement includes interest in, enthusiasm for, and investment in teaching. While some studies have explored various positive and negative affective factors influencing teachers’ engagement (Greenier et al., 2021; Derakhshan et al., 2022; Fute et al., 2022; Dai and Wang, 2023; Liu et al., 2023), limited attention has been given to the role of EFL teachers’ value congruence, mediated by enthusiasm, on work engagement.

One potentially influential factor is the concept of value congruence, which refers to the alignment between individuals’ personal values and the values espoused by their organization or work environment (Cable and DeRue, 2002). Value congruence has been found to be associated with various positive outcomes, such as job satisfaction, organizational commitment, and performance (Chatman, 1989; Byza et al., 2019; Wang and Hall, 2019; Qiao and Hu, 2021). For teachers, value congruence means the extent to which they feel their beliefs and values align with those upheld by their school (Wang and Hall, 2018; Qiao and Hu, 2021). When teachers adapt and align their beliefs and values with the school’s established norms, they can achieve higher levels of job satisfaction and develop a more professional identity (Roness, 2011; Collinson, 2012). Studies conducted on K-12 teachers in Turkey (Erdogan et al., 2004) and Norway (Skaalvik and Skaalvik, 2011) have demonstrated a positive link between perceived value congruence and job satisfaction. Teachers who experience sufficient value congruence tend to enjoy their work more and experience less burnout, benefiting from a harmonious and preferred professional environment (Siegall and McDonald, 2004). However, limited research has examined the specific role of value congruence in the context of English as a Foreign Language (EFL) teachers’ work engagement and well-being.

Furthermore, recent studies have highlighted the importance of teacher enthusiasm as a key driver of student engagement and motivation (Keller et al., 2016; Frenzel et al., 2019; Lazarides et al., 2019). Teacher enthusiasm refers to a positive affective state characterized by excitement, passion, and energy in the teaching process. It is closely related to teacher engagement and has been found to positively impact student outcomes (Sutton and Wheatley, 2003; Burić, 2019; Burić and Moe, 2020). Teacher enthusiasm, a positive emotion derived from positive psychology, is a crucial personal-professional characteristic that predicts teachers’ behavior, emotions, and success (Kunter et al., 2011; Keller et al., 2014; Lazarides et al., 2019). It is characterized as the level of gratification, enthusiasm, and delight that educators encounter in their occupational endeavors (Kunter et al., 2008). Teacher enthusiasm encompasses positive affective involvement and social presentation in teaching, nonverbal and verbal engagement in communication with others, and instructional and professional competence, among other socio-affective and cognitive traits (Kunter et al., 2013; Keller et al., 2018; Lazarides et al., 2018). This positive emotion has been shown to enhance learners’ motivation, engagement, and concentration (Zhang, 2014), as well as teachers’ performance, engagement, and fulfillment (Keller et al., 2014). Given its significant impact on teaching, teacher enthusiasm can be seen as a potential mediator between value congruence and work engagement. However, the relationship between value congruence, teacher enthusiasm, and work engagement remains relatively unexplored in the EFL teaching context.

This study aims to address this gap in the literature by examining the influence of value congruence on EFL teachers’ work engagement and well-being, with a specific focus on the mediating role of teacher enthusiasm. While previous research has established the importance of value congruence and teacher enthusiasm separately, little is known about how these factors interact and jointly influence teacher well-being. Understanding the mechanisms through which value congruence and teacher enthusiasm impact work engagement can provide valuable insights for educational institutions and policy-makers in creating supportive work environments that enhance teacher motivation and satisfaction. By investigating the relationship between value congruence, teacher enthusiasm, and work engagement in the Chinese EFL teaching context, this study aims to contribute to the existing literature on work engagement and shed light on the factors that promote teacher well-being. The findings have practical implications for educational institutions, as they can inform the development of interventions and strategies aimed at improving teaching quality, enhancing teacher job satisfaction, and ultimately benefiting both teachers and students.

## 2. Review of the literature

### 2.1. Teacher work engagement

Work engagement has been extensively studied in the field of education, leading to various models and definitions. Kahn (1990) defines it as a state of physical, cognitive, and emotional involvement in one’s profession. Similarly, Smith et al. (2012) emphasize that individuals consider themselves actively present and psychologically engaged in their work. Within the educational setting, work engagement is defined as a favorable, fulfilling, and job-related mental condition marked by immersion, commitment, and vitality (Schaufeli et al., 2002, p. 74), forming a three-dimensional model. Absorption refers to the joy and pleasure experienced while deeply engaging in work roles (Bakker and Demerouti, 2008). Dedication involves a sense of pride, inspiration, and commitment to one’s occupation (Bakker and Demerouti, 2008). Lastly, vigor entails actively and energetically putting effort into dealing with job challenges persistently (Schaufeli et al., 2002). From this perspective, engaged teachers exhibit increased concentration, dedication, and passion when teaching a foreign language to second language learners (Hakanen et al., 2006; Høigaard et al., 2012; Perera et al., 2018).

Teacher work engagement has gained significant attention due to the shift from focusing on negative emotions to positive ones, influenced by positive psychology (Burić and Macuka, 2018; Greenier et al., 2021; Zhang et al., 2023a). Unlike burnout, which can diminish happiness and efficacy (Sadeghi and Khezrlou, 2016), work engagement is seen as a potential enhancer of effectiveness and personal growth (Burić and Macuka, 2018; Dai and Wang, 2023). It holds promising implications, including increased positive emotions, greater involvement and commitment, and improved job performance (Bakker and Demerouti, 2008). In the context of language learning and teaching, EFL teachers’ work engagement is vital for fostering communicative and social involvement, impacting both teachers’ fulfillment, growth, and performance, as well as students’ active participation, academic achievement, and motivation (Han and Wang, 2021; Derakhshan et al., 2022; Xing, 2022; Li et al., 2023; Nazari et al., 2023; Zhang et al., 2023b).

Work engagement plays a vital role in shaping EFL teachers’ professional experiences, influencing their motivation, well-being, and effectiveness within the classroom (Xing, 2022). Numerous studies have delved into the factors that impact EFL teacher work engagement, shedding light on various teacher-related variables that contribute to this phenomenon. Greenier et al. (2021) conducted a study examining how emotion regulation and teacher psychological well-being influence the work engagement of British and Iranian EFL teachers. They found that both emotion regulation and teacher psychological well-being had positive effects on EFL teachers’ work engagement. However, teacher well-being emerged as the stronger predictor, underscoring its significant role in enhancing work engagement. Notably, the impact of these variables was more pronounced among British teachers, emphasizing the importance of cultural context in understanding work engagement in EFL teachers. In another study, Xiao et al. (2022) explored self-efficacy and enjoyment as predictors of teachers’ work engagement. Their findings indicated that both factors were significant predictors, with teacher self-efficacy exerting a stronger influence on work engagement compared to teaching enjoyment. This suggests that EFL teachers’ belief in their own abilities and confidence in their instructional skills play crucial roles in shaping their level of work engagement.

Also, Derakhshan et al. (2022) investigated the relationships among loving pedagogy, teaching for creativity, and work engagement among a multinational cohort of L2 teachers. Their study revealed significant connections between these variables, suggesting that a pedagogical approach focused on fostering creativity and a supportive, loving classroom environment positively impact EFL teacher work engagement. Faskhodi and Siyyari (2018) explored the relationship between work engagement and burnout among teachers. Their findings revealed a significant and negative association between the two, indicating that higher work engagement can act as a protective factor against burnout. Additionally, their study highlighted the positive influence of teaching experience on work engagement, suggesting that more experienced teachers may exhibit higher levels of work engagement in their profession. Dewaele and Li (2021) studied 2,002 Chinese EFL learners and discovered correlations between teacher enthusiasm, boredom, enjoyment, and social-behavioral learning engagement. Their findings indicated that teacher enthusiasm significantly impacted students’ engagement in the learning process. Furthermore, they found that enjoyment and boredom played a mediating role in the relationship between perceived learner social-behavioral engagement and teacher enthusiasm, suggesting that enthusiastic teaching practices positively influence students’ involvement. Building upon the significance of teacher enthusiasm, Dai and Wang (2023) revealed a positive influence of flow and proactive personality on teacher engagement. Flow, a state of complete absorption and focus in an activity, emerged as a significant factor influencing work engagement among teachers. This indicates that EFL teachers who experience flow in their teaching practices are more likely to exhibit higher levels of work engagement.

Taken together, the literature on EFL teacher work engagement underscores the importance of various teacher-related variables in influencing this critical aspect of teachers’ professional experiences. Emotion regulation, teacher psychological well-being, self-efficacy, teaching enjoyment, loving pedagogy, and enthusiasm have all been identified as pivotal factors shaping work engagement among EFL teachers. Understanding and nurturing these factors can significantly contribute to the overall well-being and effectiveness of EFL teachers in their language teaching profession.

### 2.2. Value congruence

Value congruence, which denotes the alignment between an individual’s personal values and those upheld within their work environment (Kristof, 1996; Cable and Edwards, 2004), has been recognized as a critical factor influencing employees’ attitudes, behaviors, and job-related outcomes (Schaubroeck et al., 2007; Zheng et al., 2019). Schwartz’s four-dimensional model categorizes values into self-transcendence, self-enhancement, maintenance, and openness to modification (Schwartz, 1992). Concerning teacher domains, studies have explored instructional values related to knowledge sharing, use of knowledge, autonomous learning, interpersonal skills, and active learning (Ennis and Chen, 1995; Amos and Weathington, 2008). However, an even more significant term impacting teachers’ positive emotions, well-being, engagement, and enthusiasm is value congruence itself.

Value congruence, as a component of person-environment fit and teachers’ sense of belonging in their profession, reflects the degree to which teachers feel their personal values align with the prevailing norms and values at their school (Kristof, 1996; Cable and Edwards, 2004; Edwards and Cable, 2009). Beyond personal values, teachers’ focus on value congruence can significantly influence their well-being, perseverance, job fulfillment, identification, interpersonal skills, and maintenance in the workplace (Cable and DeRue, 2002; Lee et al., 2017).

Numerous studies have shown that teachers who experience strong alignment between their personal values and the school context tend to have higher job satisfaction (Erdogan et al., 2004), work engagement (Li et al., 2015), lower burnout (Siegall and McDonald, 2004), and greater enthusiasm (Frenzel et al., 2009). As a result, a positive association between teachers’ value congruence and work engagement has been established (Sortheix et al., 2013). This relationship can be mediated by motivation and positive emotions, as teachers with significant value congruence are more motivated and engaged in their work (Ren, 2010). Moreover, personal factors such as positive emotions, social support, pleasure, and enthusiasm can play a crucial mediating role in the connection between value congruence and work engagement (Li et al., 2015; Huhtala and Feldt, 2016). Wang and Hall (2018) conducted a study involving 1,086 practicing teachers in Canada, which revealed that teachers with value congruence experience higher levels of well-being and determination. Similarly, Li et al. (2015) found a correlation between value congruence, motivation, autonomy, and engagement in their study. They also identified an indirect influence of value congruence on teachers’ work engagement, which was mediated by autonomy and motivation. Additionally, Qiao and Hu (2021) discovered that value congruence could impact teachers’ self-efficacy and commitment.

Understanding the impact of value congruence on EFL teacher work engagement can shed light on the unique challenges and opportunities faced by language educators. The alignment of personal values with the values upheld in the school environment plays a pivotal role in shaping teachers’ attitudes and behaviors toward their profession. Teachers who experience a strong sense of value congruence are more likely to be intrinsically motivated and dedicated to their teaching roles (Lee et al., 2017). This sense of alignment fosters a positive work environment, leading to higher levels of job satisfaction and a reduced likelihood of burnout (Erdogan et al., 2004; Siegall and McDonald, 2004). The sense of fulfillment derived from value congruence creates a virtuous cycle, where engaged and enthusiastic teachers are more likely to be effective in the classroom, leading to enhanced student learning outcomes (Frenzel et al., 2009). Moreover, the influence of value congruence extends beyond individual teachers to impact the overall school climate and culture. When teachers’ personal values align with the prevailing norms and values of the school, it cultivates a sense of community and cohesion among the faculty (Edwards and Cable, 2009). This shared sense of purpose and values can create a supportive and collaborative environment, where teachers feel empowered to innovate and contribute to the school’s collective mission. As a result, the school becomes a place of growth and professional development, attracting and retaining highly motivated and dedicated teachers (Cable and DeRue, 2002).

Despite the growing recognition of the importance of value congruence in shaping teacher work engagement, there is still a need for more research to fully understand its underlying mechanisms and effects in the context of EFL teaching. Although existing studies have provided valuable insights, further exploration is warranted to uncover the nuanced dynamics of value congruence and its relation to teaching enthusiasm and work engagement among language educators. This research endeavor can help inform teacher training programs and school policies aimed at promoting a positive and engaging work environment for EFL teachers, ultimately leading to enhanced language education outcomes for students. By addressing the gaps in the literature, this study aims to contribute to the growing body of knowledge on EFL teacher work engagement and pave the way for evidence-based strategies to support and empower language educators in their professional journey.

### 2.3. Teacher enthusiasm

Teacher enthusiasm has emerged as a prominent area of interest in educational research, especially due to its profound impact on students’ learning experiences and achievements (Keller et al., 2016). Rooted in the principles of positive psychology, teacher enthusiasm embodies a genuine sense of joy, excitement, and pleasure that teachers derive from their professional activities (Kunter et al., 2008; Burić, 2019). This characteristic is closely associated with intrinsic motivation, making it a crucial element of effective teaching (Kunter and Holzberger, 2014; Frenzel et al., 2019; Burić and Moe, 2020).

Over time, various definitions of teacher enthusiasm have been put forth, emphasizing its connection to teachers’ pedagogical behaviors, such as vocal delivery, gestures, facial expressions, word choice, and overall energy level (Collins, 1978; Kunter et al., 2011; Keller et al., 2016). This motivational and affective-behavioral feature of teachers is linked to their enjoyment and pleasure in teaching, making it an essential determinant of their instructional practices (Kunter et al., 2008). Teachers’ enthusiasm is communicated through both linguistic and non-linguistic behaviors, making it perceptible and understandable to students (Patrick et al., 2000; Kunter and Holzberger, 2014; Lazarides et al., 2018). Consequently, teacher enthusiasm has been found to exert a significant influence on various student outcomes, including academic achievement, interest, self-efficacy, motivation, enjoyment, and active learning (Frenzel et al., 2009; Kunter et al., 2013; Keller et al., 2014; Lazarides et al., 2018).

Notably, enthusiastic teachers are not only a source of motivation and inspiration for learners but also experience greater happiness and better overall health (Keller et al., 2016). Their active and engaged approach to teaching contributes to the creation of enjoyable and stimulating learning experiences for students (Frenzel et al., 2009; Lazarides et al., 2018, 2019). Employing humor, fun, and a social, collaborative verbal and nonverbal style, enthusiastic teachers establish strong rapport and confidence with their students (Frenzel et al., 2009; Keller et al., 2018). These qualities have been associated with enhanced foreign language teaching engagement among EFL teachers (Dewaele and Li, 2021), suggesting that higher levels of enthusiasm and enjoyment in teaching a foreign language are conducive to greater work engagement.

Furthermore, teacher enthusiasm holds the potential to act as a mediator in the relationship between value congruence and work engagement. As this study aims to explore the degree of these relationships, it seeks to contribute valuable insights into the complex interplay between teacher values, enthusiasm, and engagement in the context of EFL teaching. By investigating the mediating role of teacher enthusiasm, this research endeavors to offer a deeper understanding of the mechanisms that underlie the association between value congruence and work engagement among EFL teachers. Moreover, the impact of teacher enthusiasm extends beyond immediate student outcomes. Enthusiastic teachers often cultivate a positive classroom climate and foster a culture of active learning and student engagement (Keller et al., 2018; Frenzel et al., 2019; Burić and Moe, 2020). Students taught by enthusiastic teachers are more likely to experience a sense of belonging and emotional connection to the learning process, leading to increased motivation and interest in their studies (Keller et al., 2016; Lazarides et al., 2018). This positive classroom environment not only enhances students’ academic performance but also contributes to their overall well-being and socio-emotional development (Keller et al., 2016; Frenzel et al., 2019). Additionally, teacher enthusiasm can influence the development of students’ positive attitudes toward learning and their willingness to take on challenges and persevere in the face of difficulties (Kunter et al., 2013). Enthusiastic teachers serve as role models for curiosity, passion for learning, and the joy of discovery (Kunter et al., 2011; Lazarides et al., 2019). Such role modeling can have a lasting impact on students’ lifelong learning attitudes and behaviors, encouraging them to become self-directed, motivated learners (Kunter and Holzberger, 2014; Burić, 2019).

Taken together, teacher enthusiasm plays a pivotal role in shaping students’ learning experiences, academic achievements, and socio-emotional development. It is a vital characteristic of effective teaching, closely linked to intrinsic motivation and the creation of a positive classroom climate. Enthusiastic teachers inspire and motivate their students, fostering a love for learning and a sense of belonging in the classroom. Additionally, teacher enthusiasm has the potential to act as a mediating factor in the relationship between value congruence and work engagement among EFL teachers. Understanding the impact of teacher enthusiasm on work engagement can provide valuable insights into the mechanisms that drive teachers’ engagement and satisfaction in their profession. This study aims to contribute to the existing body of knowledge by exploring the interconnectedness of value congruence, teacher enthusiasm, and work engagement among EFL teachers, shedding light on ways to enhance teacher well-being and effectiveness in the classroom.

### 2.4. The present research

The present study proposes a hypothesized model to investigate the relationships between value congruence, teaching enthusiasm, and work engagement among EFL teachers. The following hypotheses are justified based on the existing literature:

*H1*: Teachers who perceive a higher level of value congruence with their school will have higher levels of work engagement than teachers who perceive a lower level of value congruence.

This hypothesis is grounded in the theory that value congruence, which refers to the alignment between an individual’s personal values and the values upheld within their work environment, positively influences job-related outcomes and attitudes (Schaubroeck et al., 2007; Zheng et al., 2019). Teachers who perceive a strong alignment between their personal values and the values espoused by their educational institution are more likely to experience a sense of purpose, commitment, and enthusiasm in their work (Li et al., 2015; Huhtala and Feldt, 2016). Work engagement involves being emotionally, cognitively, and physically involved in one’s profession (Kahn, 1990; Perera et al., 2018). Thus, when teachers perceive a higher level of value congruence, they are more likely to be emotionally and cognitively engaged in their teaching, leading to higher levels of work engagement (Wang and Hall, 2019; Qiao and Hu, 2021; Fute et al., 2022).

*H2*: Teaching enthusiasm will mediate the relationship between value congruence and work engagement, such that teachers who perceive a higher level of value congruence will have higher levels of teaching enthusiasm, which, in turn, will lead to higher levels of work engagement.

This hypothesis is based on the principles of positive psychology, which emphasize the role of positive affect and emotions in driving engagement and motivation (Fredrickson, 2001; Sutton and Wheatley, 2003). When teachers perceive a strong alignment between their values and those of their educational institution, they are more likely to experience higher levels of enthusiasm in their teaching (Donnell and Gettinger, 2015; Wang and Hall, 2019). Teacher enthusiasm, characterized by passion, excitement, and energy, is closely associated with intrinsic motivation and serves as a key driver of work engagement (Kunter et al., 2011; Li et al., 2015). Thus, teaching enthusiasm is expected to mediate the relationship between value congruence and work engagement, as teachers who perceive higher levels of value congruence will experience greater enthusiasm in their teaching, leading to higher levels of work engagement.

*H3*: The effect of value congruence on work engagement will be stronger for teachers with higher levels of teaching enthusiasm than for teachers with lower levels of teaching enthusiasm.

This hypothesis posits that teaching enthusiasm moderates the relationship between value congruence and work engagement. The motivational and affective-behavioral characteristics of teaching enthusiasm are closely linked to teachers’ enjoyment and pleasure in teaching (Kunter et al., 2008). Teachers who experience high levels of enthusiasm are more likely to be emotionally and cognitively engaged in their work, amplifying the positive impact of value congruence on work engagement (Keller et al., 2014). Consequently, for teachers with higher levels of teaching enthusiasm, the alignment between their personal values and the values of their educational institution is expected to have a stronger positive effect on their work engagement compared to teachers with lower levels of teaching enthusiasm. This moderating effect of teaching enthusiasm on the relationship between value congruence and work engagement underscores the importance of understanding the interplay between individual characteristics and contextual factors in shaping teachers’ engagement in their profession.

## 3. Method

### 3.1. Participants

Researchers in Mainland China sent out study invitations to 640 teachers in the classroom setting. Out of these invitations, 453 valid questionnaires were received, resulting in a response rate of 70.78%. Among the participants, 206 were male (45.45%) and 247 were female (54.55%). The age range of the participants was from 21 to 48 years, with an average age of 28.32 (SD = 7.83). On average, the teachers had served in schools for approximately 9.23 years (SD = 9.63).

The participants were recruited from diverse educational settings, including primary schools, secondary schools, and universities. They represented a range of subject areas, including language arts, mathematics, science, and social studies. The teachers’ experience levels varied, with some participants being early-career teachers while others had been teaching for several decades.

Informed consent was obtained from all participants before they completed the measures. The study followed ethical guidelines ensuring the confidentiality and anonymity of participants. The researchers emphasized that participation was voluntary and would not have any impact on the participants’ employment or professional evaluations. Participants were assured that their responses would be kept strictly confidential and only used for research purposes.

To minimize bias, participants were instructed to provide honest and accurate responses to the questionnaire items. The questionnaire took approximately 15–25 min to complete, and participants were given the flexibility to choose a convenient time and location for completing the survey.

### 3.2. Measures

#### 3.2.1. Teacher enthusiasm

To assess enthusiasm while teaching, a selection of 5 items from the Teacher Enthusiasm Scale (Kunter et al., 2011), as adapted by Burić and Moe (2020), was employed. Teachers were instructed to indicate their agreement with each item using a 5-point rating scale, where 1 represented “strongly disagree” and 5 represented “strongly agree.” An illustrative item from the scale was: “I teach with great enthusiasm.”

#### 3.2.2. Value congruence

The scale used to measure value congruence among teachers in this study was a modified version of Cable and DeRue’s (2002) perceived value congruence scale. The scale consisted of three items that assessed the alignment between the teachers’ personal values and the values upheld by their school. Participants rated their agreement with statements such as “The things that I value in life are very similar to the things that my school values.” Responses were provided on a 5-point Likert scale, ranging from “1 = strongly disagree” to “5 = strongly agree.”

#### 3.2.3. Work engagement

The assessment of teachers’ work engagement in this study utilized a modified version of the three-dimensional scale developed by Li et al. (2006), which was originally derived from the Utrecht Work Engagement Scale (Schaufeli et al., 2002). Participants were asked to rate their responses to all items using a 5-point Likert scale, ranging from “1 = strongly disagree” to “5 = strongly agree.” The scale consisted of 16 items, with each dimension encompassing a different number of items: specifically, the vigor (VI) dimension comprised 6 items, the dedication (DE) dimension had 5 items, and the absorption (AB) dimension included 5 items. An illustrative item from the scale was “When I get up in the morning, I feel like going to school.”

### 3.3. Procedure

The data for this study were collected through an online survey administered to teachers in Mainland China. To ensure a comprehensive and efficient data collection process, a systematic approach was employed. Specifically, an online survey platform was utilized, enabling easy distribution, data collection, and management of responses. Invitations were sent via email to potential participants, who were teachers from various educational institutions across Mainland China. These invitations provided a brief overview of the research objectives and emphasized the importance of the teachers’ contribution to the study.

Prior to accessing the online survey, participants were presented with an informed consent form. They were required to carefully read and understand the purpose of the study, the voluntary nature of their participation, the confidentiality of their responses, and their rights as participants. Only those who provided their informed consent were granted access to the questionnaire.

The online questionnaire was thoughtfully developed, taking into account the constructs of interest, such as perceived teacher caring, student wellbeing, and other relevant variables. It underwent a rigorous process of review, ensuring clarity and relevance. Additionally, it was pilot tested prior to the data collection phase to further enhance its quality. The questionnaire was designed to be user-friendly and accessible on various devices, making it convenient for participants to complete.

The data collection period lasted for 6 weeks, allowing participants ample time to complete the questionnaire at their convenience. Throughout this period, reminders and follow-up emails were sent to encourage participation and maximize the response rate. The researchers approached teachers respectfully and professionally during the data collection process. They emphasized the confidential nature of the study and assured participants that their responses would be anonymized and used solely for research purposes. Measures were implemented to safeguard the privacy and confidentiality of participants’ personal information.

To ensure data quality, data monitoring and quality control procedures were in place. The researchers carefully monitored the survey responses, conducting checks to identify and address any duplicate or inconsistent responses. In case participants had any questions or concerns regarding the survey, contact information was provided for them to seek clarification.

Ethical considerations were of utmost importance in this study. The research strictly adhered to ethical guidelines, which included obtaining informed consent, ensuring participant confidentiality, and protecting the rights and well-being of the participants. The research protocol underwent a thorough review and was approved by the relevant institutional ethics committee.

### 3.4. Data analysis

Data analysis was conducted using SPSS (Version 28) and Amos (Version 28) with Maximum Likelihood Estimation (MLE). To test the relationships among the constructs, structural equation modeling (SEM) was employed. Prior to running SEM, the measurement model was evaluated through confirmatory factor analysis (CFA) to ensure the construct validity of the scales. The fit indices used to assess the model fit included the *χ*^2^-goodness of fit to the degree of freedom (df) ratio, the Goodness of Fit Index (GFI), the Comparative Fit Index (CFI), the Root-Mean-Square Error of Approximation (RMSEA), and the Standardized Root-Mean-Square Residual (SRMR).

For the *χ*^2^/df ratio, a value less than 3 and a value of p greater than 0.05 were considered indicative of a good fit (Marsh et al., 2004). Additionally, fit indices such as GFI and CFI values of 0.90 or higher were used to determine a good fit. Moreover, RMSEA values below 0.08 and SRMR values below 0.10 were considered as thresholds for good fit (Marsh et al., 2004; Kline, 2023).

## 4. Results

Preliminary analyses were conducted to ensure the quality and integrity of the data. Several aspects were examined, including missing data, normality, and outliers, following the recommendations of Kline (2023). To address missing data, the Expectation–Maximization (EM) algorithm was employed in this study, as it is a suitable technique for imputing missing values, particularly in studies with small sample sizes and a high number of missing data (Tabachnick et al., 2013). The normality of the items was assessed by examining the skewness and kurtosis indices. Items with skewness and kurtosis values exceeding ±2.0 were considered to deviate from a normal distribution and were subsequently identified and removed from the analysis (Kline, 2023). Additionally, both univariate and multivariate outliers were examined. Univariate outliers were detected using Z-standardized scores, while multivariate outliers were identified using the Mahalanobis $D^{2}$ distance measure (Tabachnick et al., 2013; Kline, 2023). Any identified outliers were subsequently removed from the dataset. Following these data screening procedures, the final sample consisted of 446 participants, ensuring a robust and reliable dataset for further analyses.

After that, the construct validity of the measures employed in the study was examined to ensure their adequacy in assessing the targeted constructs. Table 1 presents the outcomes of the first-order Confirmatory Factor Analyses (CFAs) along with the reliability indices for the three measurement scales used in the research. The results demonstrate the satisfactory goodness of fit for each scale, affirming their suitability for capturing the intended constructs accurately. Moreover, the reliability coefficients provide evidence of the internal consistency of the scales, further establishing their reliability in measuring the variables of interest. Together, these findings support the construct validity and reliability of the utilized measures in this study.

**Table 1:** CFA results.

	CMIN	DF	CMIN/DF	*P*	CFI	RMSEA	SRMR	α
Value congruence	185.241	96	1.92	<0.001	0.961	0.056	0.047	0.79
Teacher enthusiasm	281.358	154	1.82	<0.001	0.976	0.043	0.039	0.83
Work engagement	364.201	194	1.87	<0.001	0.983	0.038	0.033	0.87

Table 2 displays the descriptive statistics and correlations among the constructs examined in the study. The first construct, value congruence, exhibits a mean of 4.04 (SD = 0.93) and shows a significant correlation with enthusiasm (*r* = 0.46, *p* < 0.01) and work engagement (*r* = 0.29, *p* < 0.05). On the other hand, enthusiasm demonstrates a mean of 3.27 (SD = 0.81) and shows a positive correlation with both value congruence (*r* = 0.46, *p* < 0.01) and work engagement (*r* = 0.48, *p* < 0.01).

**Table 2:** Descriptive statistics and correlations between the constructs.

Constructs	1	2	3
1. Value congruence	1		
2. Enthusiasm	0.46**	1	
3. Work engagement	0.29*	0.48**	1
4. Mean	4.04	3.27	3.91
5. SD	0.93	0.81	0.78
6. Skewedness	−0.36	−0.32	−0.26
7. Kurtosis	−0.42	−0.51	−0.38

^*^p < 0.05.

^**^p < 0.01.

Lastly, the third construct, work engagement, displays a mean of 3.91 (SD = 0.78) and is positively correlated with both value congruence (*r* = 0.29, *p* < 0.05) and enthusiasm (*r* = 0.48, *p* < 0.01).

Table 3 displays the comparative analysis of male and female students on the variables of value congruence, teacher enthusiasm, and work engagement. For value congruence, male students obtained a mean score of 3.89 (SD = 0.89), while female students had a slightly higher mean score of 4.12 (SD = 0.91). However, the results of the t-test indicated that the difference in value congruence between males and females was not statistically significant (*t* = −0.549, *p* = 0.376).

**Table 3:** A comparative analysis of male and female students.

	Males	Females	*t*-value	value of *p*
Variables	*M* (SD)	*M* (SD)		
Value congruence	3.89 (0.89)	4.12 (0.91)	−0.549	0.376
Teacher enthusiasm	3.20 (0.79)	3.28 (0.91)	−0.317	0.514
Work engagement	4.06 (0.88)	3.98 (0.93)	−0.248	0.418

Likewise, for teacher enthusiasm, male students had a mean score of 3.20 (SD = 0.79), and female students had a slightly higher mean score of 3.28 (SD = 0.91). The *t*-test analysis revealed no significant difference in teacher enthusiasm between males and females (*t* = −0.317, *p* = 0.514).

Regarding work engagement, male students had a mean score of 4.06 (*SD* = 0.88), while female students had a slightly lower mean score of 3.98 (*SD* = 0.93). However, the *t*-test results indicated no significant difference in work engagement between males and females (*t* = −0.248, *p* = 0.418). Overall, the findings from the comparative analysis suggest that there are no significant gender differences in the levels of value congruence, teacher enthusiasm, and work engagement among the participants.

**Figure 1:**
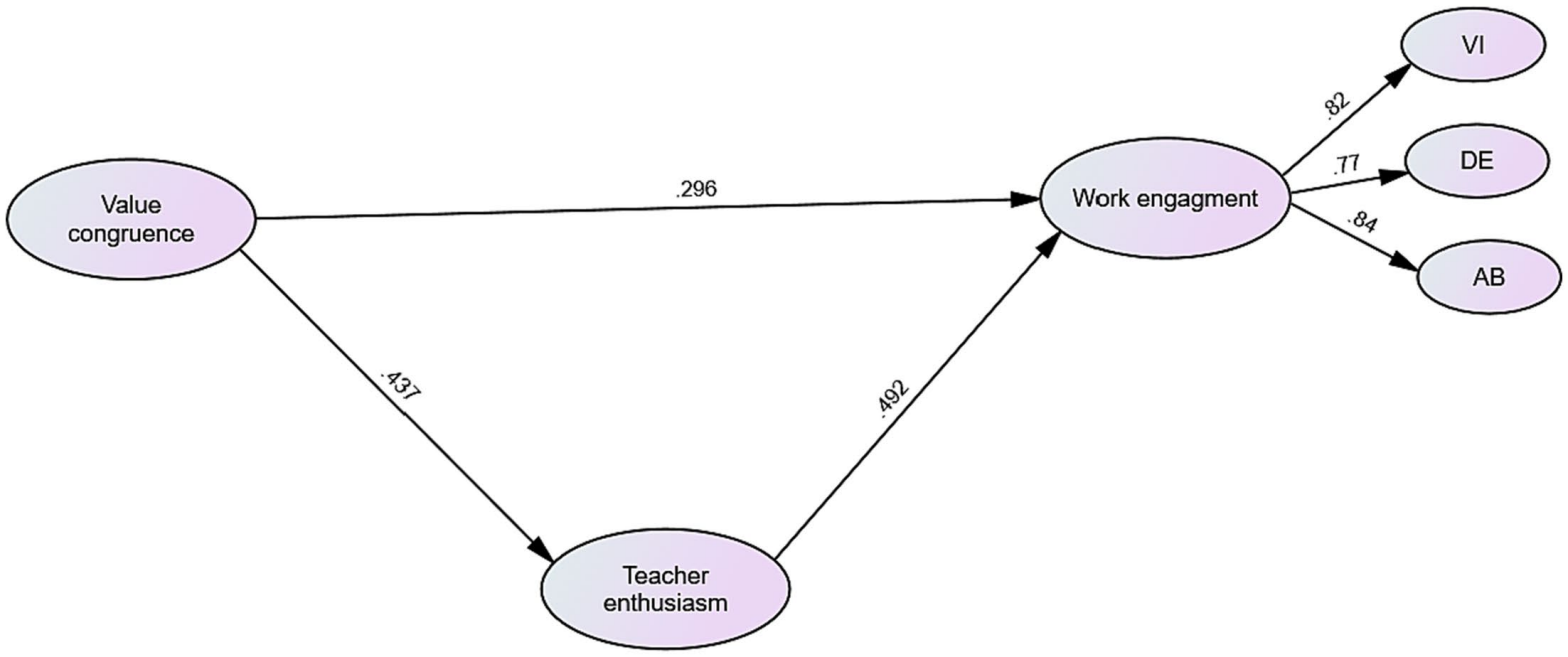
The final mediation model.

Following that, SEM was employed to investigate the potential mediating role of teacher enthusiasm in the relationship between value congruence and work engagement. The analysis indicated that the proposed model exhibited a favorable fit to the data, as evidenced by the following fit indices: *χ*^2^/df = 1.650, CFI = 0.948, TLI = 0.945, RMSEA = 0.038, and SRMR = 0.043. Figure 1 presents the standardized parameter estimates for the model proposed in this study. Then, to assess the indirect effects, a resampling technique called bootstrap resampling was employed, utilizing 500 iterations. Bootstrap resampling is a well-established approach in SEM that enables the examination of the sampling distribution and facilitates the estimation of indirect effects (Hayes, 2009).

Table 4 displays the direct and indirect effects of the structural model. The model pathways examined the relationships between value congruence, teacher enthusiasm, and work engagement. Regarding the direct effects, the path from value congruence to work engagement had a significant positive effect (*B* = 0.358, SE = 0.149, *B* = 0.296, *p* < 0.001) with a 95% confidence interval ranging from 0.067 to 0.649. Similarly, the path from value congruence to teacher enthusiasm showed a significant positive effect (*B* = 0.605, SE = 0.157, *B* = 0.437, *p* < 0.001) with a 95% confidence interval ranging from 0.297 to 0.913. Additionally, the path from teacher enthusiasm to work engagement had a significant positive effect (*B* = 0.427, SE = 0.088, *Β* = 0.492, *p* < 0.001) with a 95% confidence interval ranging from 0.255 to 0.599. In terms of the indirect effect, the path from value congruence to teacher enthusiasm to work engagement revealed a significant positive effect (*B* = 0.326, SE = 0.079, *B* = 0.215, *p* < 0.001) with a 95% confidence interval ranging from 0.130 to 0.401.

**Table 4:** Direct and indirect effects.

					95% CI	
Model pathways	*B*	SE	*Β*	*P*	Lower bound	Upper bound
Direct effects						
Value congruence → WE	0.358	0.149	0.296	<0.001	0.067	0.649
Value congruence → enthusiasm	0.605	0.157	0.437	<0.001	0.297	0.913
Enthusiasm→ WE	0.427	0.088	0.492	<0.001	0.255	0.599
Indirect effect						
Value congruence → enthusiasm→ WE	0.326	0.079	0.215	<0.001	0.130	0.401

WE, work engagement; enthusiasm, teacher enthusiasm.

These results indicate that value congruence has both direct and indirect effects on work engagement. Value congruence positively influences both teacher enthusiasm and work engagement. Additionally, teacher enthusiasm has a direct positive effect on work engagement. The indirect effect analysis demonstrates that teacher enthusiasm mediates the relationship between value congruence and work engagement. Collectively, the variables of value congruence and teacher enthusiasm accounted for 45.12% of the variance observed in work engagement, while the remaining variance was attributed to external factors.

Moreover, the researchers evaluated the structural equivalence of the mediation model to investigate whether the structural path coefficients differed significantly between male and female participants. The results revealed that the proposed model exhibited a favorable fit to the data for both male and female groups. A multi-group invariance analysis conducted across gender indicated that both the constrained model (*χ*^2^/df = 1.764, CFI = 0.962, TLI = 0.938, RMSEA = 0.040, SRMR = 0.072) and the unconstrained model (*χ*^2^/df = 1.758, CFI = 0.964, TLI = 0.935, RMSEA = 0.039, SRMR = 0.071) demonstrated a satisfactory fit to the data (see Table 5). The *χ*^2^ difference test conducted between the constrained and unconstrained models (Δ*χ*^2^ = 3.452, Δdf = 3, *p* = 0.321) indicated that the model coefficients within the proposed mediation model remained consistent across gender.

**Table 5:** Structural invariance analysis results.

Model	*χ*^2^/df	CFI	TLI	RMSEA	SRMR
Constrained model	1.764	0.962	0.938	0.040	0.072
Unconstrained model	1.758	0.964	0.935	0.039	0.071

Furthermore, we conducted separate structural equation modeling (SEM) analyses for male and female participants to examine the structural model. The results revealed that both models exhibited satisfactory fit for male participants (*χ*^2^/df = 1.860, *p* < 0.001, CFI = 0.938, RMSEA = 0.049, SRMR = 0.065) and female participants (*χ*^2^/df = 1.765, *p* < 0.001, CFI = 0.943, RMSEA = 0.042, SRMR = 0.059). These findings indicate that there were no statistically significant distinctions between male and female learners concerning both the direct and indirect impacts of teacher value congruence on work engagement. Moreover, the mediating role of teacher enthusiasm remained consistent and robust across both genders.

## 5. Discussion

The present study aimed to examine the influence of value congruence and teacher enthusiasm on Chinese EFL teachers’ work engagement and well-being. The findings provide important insights into the relationships among these constructs and contribute to our understanding of factors that promote teacher motivation and satisfaction in the EFL teaching context.

Firstly, the results revealed a significant positive relationship between value congruence and work engagement. The significant positive relationship between value congruence and work engagement found in the present study adds to the growing body of literature emphasizing the importance of this alignment in promoting job satisfaction and commitment among teachers. Consistent with previous research (Amos and Weathington, 2008; Byza et al., 2019; Wang and Hall, 2019; Qiao and Hu, 2021), our findings suggest that when teachers perceive a strong match between their personal values and the values of their organization or work environment, they are more likely to experience a sense of purpose and dedication in their work. This sense of alignment and congruence with the organizational values leads to increased enthusiasm and commitment to their profession (Li et al., 2015; Huhtala and Feldt, 2016). The psychological need satisfaction and intrinsic motivation derived from working in congruence with one’s core values (Deci and Ryan, 2000) foster a deeper emotional and cognitive engagement with their teaching responsibilities.

Work engagement, characterized by emotional, cognitive, and physical involvement in one’s profession (Kahn, 1990; Perera et al., 2018), is a critical aspect of effective teaching. Our study highlights the importance of providing teachers with support and opportunities for emotional and cognitive engagement to enhance their overall work engagement. By nurturing value congruence, which bridges personal values with the values of the work environment (Kristof, 1996; Cable and Edwards, 2004; Wang and Hall, 2019), educational institutions can promote a greater sense of belonging and cognitive alignment among teachers (Wang and Hall, 2019; Qiao and Hu, 2021; Fute et al., 2022).

The concept of cognitive engagement, as explored in this study, refers to the extent to which teachers perceive their personal values aligning with the prevailing norms and values at their school (Skaalvik and Skaalvik, 2015; Eldor and Shoshani, 2016). This cognitive alignment plays a crucial role in influencing teachers’ well-being and professional outcomes. Teachers who demonstrate adaptability and alignment of their beliefs and values with the established values of the school exhibit higher levels of cognitive engagement and tend to experience more positive outcomes in their work (Roness, 2011; Collinson, 2012; Choochom, 2016). Moreover, teachers with value congruence are more likely to display characteristics such as flexibility in their beliefs and attitudes, taking ownership of reflective practices, demonstrating autonomy in their values and performance, and engaging in both individual and collective efforts (Rothmann and Hamukang’andu, 2013; Li et al., 2015; Qiao and Hu, 2021). Such characteristics contribute to a more dynamic and fulfilling teaching experience and may ultimately lead to greater work engagement and job satisfaction.

Also, the findings of this study provide valuable insights into the mediating role of teacher enthusiasm in the relationship between value congruence and work engagement among EFL teachers. This finding aligns with theoretical frameworks emphasizing the significance of positive affect in driving engagement and motivation (Fredrickson, 2001; Sutton and Wheatley, 2003). As teachers perceive a strong alignment between their personal values and the values upheld by their educational institution, they are more likely to experience heightened levels of enthusiasm in their teaching practices (Donnell and Gettinger, 2015; Wang and Hall, 2019). This enthusiasm, characterized by excitement, passion, and energy, then becomes a crucial catalyst for their work engagement (Kunter et al., 2011).

The indirect effect of value congruence on teachers’ work engagement, as mediated by autonomy, motivation, and enthusiasm, had been highlighted in previous research by Li et al. (2015). Additionally, other studies have demonstrated that value congruence can positively influence teachers’ self-efficacy and commitment, both of which are closely related to enthusiasm (Ford et al., 2017; Shaukat et al., 2019; Qiao and Hu, 2021). Moreover, investigations have reported a positive association between perceived value congruence and job satisfaction (Erdogan et al., 2004; Skaalvik and Skaalvik, 2011; Choochom, 2016). Teachers with high levels of value congruence tend to experience greater job satisfaction and reduced burnout, owing to their alignment with a pleasant and desirable professional environment (Siegall and McDonald, 2004; Klassen et al., 2010; Donnell and Gettinger, 2015). Consequently, the positive prediction of teaching enthusiasm from value congruence significantly influences EFL teachers’ work engagement.

Furthermore, the results indicated that teacher enthusiasm fully mediates the relationship between value congruence and work engagement. This suggests that the positive impact of value congruence on work engagement operates through the mechanism of teacher enthusiasm. These findings are in line with the broaden-and-build theory of positive emotions (Fredrickson, 2001; Greenier et al., 2021; Dai and Wang, 2023), which posits that positive affective experiences, such as enthusiasm, broaden individuals’ cognitive and behavioral repertoires, leading to increased engagement and well-being.

Work engagement, defined as a persistent, positive, affective-motivational state of fulfillment involving vigor, dedication, and absorption (Maslach et al., 2008), is conceptually related to teachers’ interest in, enthusiasm for, and investment in teaching (Cardwell, 2011). Enthusiasm and absorption serve as primary components of teachers’ work engagement, reflecting the joy and pleasure they experience in the teaching process (Bakker and Demerouti, 2008). Teacher enthusiasm refers to the level of delight, exhilaration, and satisfaction that teachers commonly encounter in their professional endeavors (Kunter et al., 2008). It is a comprehensive demonstration of positive emotional involvement in a socio-affective and cognitive context (Keller et al., 2018), encompassing both nonverbal and verbal involvement in communication with others (Dewaele and Li, 2021).

Therefore, when combined with value congruence, enthusiasm enhances teachers’ work engagement, as teachers with value congruence are more cognitively and emotionally engaged, leading to greater enthusiasm (Keller et al., 2014). This suggests that fostering value congruence and promoting a positive and enthusiastic teaching environment can be effective strategies for enhancing EFL teachers’ work engagement and overall well-being. By understanding the mediating role of teacher enthusiasm, educational institutions can design targeted interventions to improve teacher motivation and satisfaction, ultimately contributing to the quality of language education and students’ learning experiences.

## 6. Conclusion

The present study represents a comprehensive investigation into the influence of value congruence and teacher enthusiasm on EFL teachers’ work engagement and well-being. The findings contribute significantly to the existing literature by shedding light on the significance of these factors and their interplay in promoting teacher motivation and satisfaction within the context of EFL teaching. The study yields valuable insights and implications that can be of great interest to educational institutions, policymakers, and practitioners alike.

Firstly, the study underscores the pivotal role of value congruence in shaping EFL teachers’ work engagement. The results reveal that when teachers perceive a strong alignment between their personal values and the values espoused by their educational institution, they are more likely to experience higher levels of work engagement. This underscores the importance of fostering an organizational environment that nurtures and sustains value congruence among teachers. Educational institutions can proactively create shared values, promote open channels of communication, and involve teachers in decision-making processes to enhance value congruence and consequently elevate work engagement levels. Secondly, the study substantiates the mediating role of teacher enthusiasm in the relationship between value congruence and work engagement. Teacher enthusiasm, characterized by passion, excitement, and energy, serves as a critical mechanism through which value congruence positively influences work engagement. As a result, educational institutions should prioritize efforts to cultivate and sustain teacher enthusiasm. Implementing professional development opportunities, recognizing and celebrating teachers’ achievements, and fostering a positive and supportive work climate are effective strategies to enhance teacher enthusiasm and subsequently promote work engagement.

Furthermore, the study reveals no significant gender differences in the relationships examined. Both male and female teachers exhibited similar levels of work engagement and well-being. These findings suggest that interventions and strategies aimed at enhancing work engagement and well-being can be implemented regardless of gender. However, it is essential to acknowledge the potential presence of other contextual factors that may influence these relationships. Future research could explore additional factors or moderators that may contribute to gender differences in work engagement and well-being among EFL teachers. The implications of this study extend beyond theoretical insights. Educational institutions can greatly benefit from the findings by adopting evidence-based practices to enhance teacher motivation and job satisfaction. By fostering value congruence, promoting teacher enthusiasm, and creating a supportive work environment, educational institutions can not only improve teacher well-being but also enhance the overall quality of education provided to students. Engaged and motivated teachers are more likely to be effective in the classroom, leading to better student outcomes. Moreover, policy implications arise from this study. Policymakers should recognize the importance of valuing and supporting teachers’ personal values in educational settings. They should prioritize policies that cultivate a positive work environment and provide teachers with opportunities for professional growth and development. By acknowledging the significance of teacher motivation and well-being, policymakers can contribute to the overall improvement of the education system and positively impact the teaching-learning process.

Despite the valuable contributions of this study, it is essential to recognize several limitations that may impact the interpretation and generalization of the findings. Firstly, the study relied on self-report measures, which may be susceptible to common method bias and social desirability bias. While measures were taken to ensure anonymity and confidentiality, participants’ responses might have been influenced by their perceptions of socially desirable answers. To enhance the validity of future research, the inclusion of multiple data sources and objective measures could mitigate these potential biases. Secondly, the study was conducted within a specific context of Chinese EFL teachers, which may restrict the generalizability of the results to other educational settings or teacher populations. Cultural, institutional, and contextual factors may interact differently with the examined relationships in diverse educational contexts. Conducting replications of the study in varied settings and with different teacher populations would provide a more comprehensive understanding of the phenomenon under investigation.

Thirdly, the cross-sectional design of the study precludes the establishment of causal relationships. As the findings are based on observed associations at a particular moment in time, longitudinal or experimental designs are needed to ascertain the temporal sequence and causal links between the variables. Future research employing longitudinal studies would offer valuable insights into the dynamics of value congruence, teacher enthusiasm, work engagement, and well-being over time. Additionally, the study focused on a specific set of variables, namely value congruence, teacher enthusiasm, work engagement, and well-being. Other factors that potentially contribute to teacher motivation and satisfaction were not included in the analysis. Future research should explore the role of additional variables, such as organizational support, job autonomy, and teacher-student relationships, to attain a more comprehensive understanding of teacher motivation and well-being. Lastly, the study exclusively employed quantitative analysis and did not incorporate qualitative approaches to capture a deeper understanding of teachers’ experiences and perceptions. Qualitative methods, such as interviews or focus groups, could provide rich insights into the underlying mechanisms and contextual factors influencing teacher motivation and well-being. Integrating both quantitative and qualitative approaches would yield a more comprehensive and nuanced understanding of the research topic.

## Data availability statement

The data analyzed in this study is subject to the following licenses/restrictions: the data supporting the conclusions of this article will are available by the authors, without undue reservation. Requests to access these datasets should be directed to WZ, Email: jingzw@hotmail.com.

## Ethics statement

The studies involving humans were approved by the Department of Humanities and Law, Hebei University of Engineering, Handan 056038, Hebei, China. The studies were conducted in accordance with the local legislation and institutional requirements. The participants provided their written informed consent to participate in this study.

## Author contributions

JC: Conceptualization, Formal analysis, Funding acquisition, Methodology, Project administration, Resources, Software, Supervision, Visualization, Writing – original draft, Writing – review & editing. WZ: Conceptualization, Data curation, Funding acquisition, Investigation, Methodology, Project administration, Resources, Software, Validation, Visualization, Writing – original draft, Writing – review & editing.
